# Diagnosis of Death

**Published:** 1875-03

**Authors:** 


DIAGNOSIS OF DEATH.—Dr. Monteverdi, of Cremona, has proposed a simple, easy and certain method of seeing whether a person is really dead. He injects a drop or two of ammonia beneath the skin. If the person be dead, no effect, or next to none, is produced; but if the person be alive, a red color appears at the point of injection.—*Phila. Med. Times, 9th Jan.*, 1875.

